# In Situ Synthesis of Al-Based MMCs Reinforced with AlN by Mechanical Alloying under NH_3_ Gas

**DOI:** 10.3390/ma11050823

**Published:** 2018-05-17

**Authors:** E. S. Caballero, F. G. Cuevas, F. Ternero, R. Astacio, J. M. Montes, J. Cintas

**Affiliations:** 1 Escuela Técnica Superior de Ingeniería, Universidad de Sevilla, Camino de los Descubrimientos, s/n. 41092 Sevilla, Spain; fternero@us.es (F.T.); rastacio@us.es (R.A.); jmontes@us.es (J.M.M.); jcintas@us.es (J.C.); 2 Escuela Técnica Superior de Ingeniería, Universidad de Huelva, Campus El Carmen, Avda. 3 de marzo, s/n. 21071 Huelva, Spain; fgcuevas@dqcm.uhu.es

**Keywords:** aluminum, AMCs, powder metallurgy, mechanochemical processing, mechanical properties

## Abstract

Aluminum matrix composites (AMCs) reinforced by aluminum nitride were prepared by mechanical alloying followed by a simple press and sintering method. Milling began under vacuum and after a period of between 1 and 4 h, NH_3_ gas flow (1 cm^3^/s) was incorporated until the total milling time of 5 h was reached. Results show that in addition to the strain hardening taking place during mechanical alloying, NH_3_ plays an additional role in powder hardening. Thereby, the properties of the sintered compacts are strongly influenced by the amount of N incorporated into the powders during milling and the subsequent formation of AlN during the consolidation process. The obtained AMC reaches tensile strengths as high as 459 MPa and hardness much higher than that of the as-received aluminum compact.

## 1. Introduction

Because of their low weight and moderate strength, aluminum alloys are of great interest for a wide range of structural applications, especially in the aerospace and automotive industries [1,2]. However, the increasing demand for a high specific strength and properties stability at elevated temperatures [3,4] make necessary the development of new aluminum alloys. The improvement of these properties can be achieved through new aluminum matrix composites (AMCs) [5,6,7], which are based in an aluminum matrix reinforced by ceramic particles. Because of their excellent properties, AMCs are nowadays widely used in sectors such as transportation, aerospace, electronics, sports, and infrastructure industries [8,9]. Traditionally, SiC and Al_2_O_3_ particles have been mainly used as reinforcement phases [10,11,12,13,14], but the desired performance improvements have led to reinforcing with many other types of ceramic particles (Si_3_N_4_, AlN, B_4_C, TiC, ZrB_2_, TiB_2_). There are several methods by which to produce AMCs, including stirring casting [15], pressure infiltration [7,10,16], spray deposition, accumulating roll bonding [17], centrifugal casting [18], and powder metallurgy [19]. 

MMC (metal matrix composite) properties can be controlled by using different types, amounts, sizes, and morphologies of the reinforcement, it being very important for these particles to be homogeneously distributed in the matrix. Over the last few years, studies have been conducted to achieve a good distribution of the reinforcing particles, as well as the optimal reinforcement–matrix volume ratio [13]. One of the manufacturing processes allowing the reaching of these aims is mechanical alloying (MA), with continuous fracture and welding processes taking place between the matrix and reinforcements incorporated into the mill, producing the desired microstructure. In addition, if chemical reactions are activated during milling (mechanosynthesis), the reinforcing phases can be obtained in situ or in a subsequent heating process, producing an even better homogeneous distribution of the reinforcements [20]. In these cases, the main challenge is to generate the appropriate reinforcement phases, and control both their size and percentage.

The aim of this work is to produce AMCs reinforced with a self-forming nitride dispersion. To this end, the aluminum-matrix powder was processed in a high-energy ball mill, and an ammonia gas flow was incorporated to produce a gas–solid reaction. The weight percentage of the nitrogen-rich phases was controlled by using different NH_3_ gas flow times. Thus, a combination of different sequences of vacuum and ammonia gas was carried out for a total milling time of five hours. After the milling process, powders were analyzed and compacts were made from them to study their mechanical properties.

## 2. Materials and Experimental Procedure

The starting material was atomized elemental aluminum powder (AS 61, Eckart) with a purity level higher than 99.7% and a mean particle size of 80.5 μm. The Al powder was processed in a high-energy attritor ball mill. The water-cooled stainless steel vessel had a capacity of 1400 cm^3^. A 3 wt % ethylene bis-stearamide organic wax (EBS, Licowax^®^ C micro powder PM Clariant, Basel, Switzerland) was used to balance the welding and fracture processes of the Al powders during milling. The mill contained 72 g of powder and 3600 g of high-chromium steel balls (charge ratio 50:1). All milling experiments were performed with a rotor speed of 500 rpm, at room temperature. In order to study the influence of the NH_3_ milling time on the formation of nitrogen-rich phase reinforcements, the ammonia flow period was extended from 1 to 4 h. As a layer of Al_2_O_3_ covers the aluminum particles, all the experiments started under vacuum with the aim of eliminating and improving the reactivity of such particles with the NH_3_ gas. Thus, the experiments started under vacuum, and after a period of between 1 and 4 h, ammonia (NH_3_) gas flow (1 cm^3^/s, purity > 99.96%, Air Liquide) was incorporated until the total milling time of 5 h was reached [21]. Table 1 summarizes the milling time under vacuum and NH_3_ gas flow for the different milling experiments. Additionally, the effect of using a shorter ammonia flow, in particular for 10 min (10’A), was also studied. In this case, ammonia flow was incorporated after 2 h of milling, and then millings continued under vacuum until reaching 5 h.

All milled powders were consolidated by uniaxial cold pressing at 850 MPa and vacuum sintering (5 Pa) at 650 °C for 1 h, followed by furnace cooling. For comparison purposes, as-received aluminum powder (AR Al) was also consolidated by the same process. EBS wax was also used as the die-wall lubricant during cold pressing. Both cylindrical- (diameter: 12 mm; mass: 4 g) and tensile-shaped specimens [22] were produced.

A universal testing machine (Instron 5505, Instron, Norwood, MA, USA) with a load cell of 100 kN was used to evaluate the compressibility of the powders. X-ray diffraction analysis (XRD, Bruker D8 Advance, using CuKα radiation, step size of 0.015°, and time step of 0.5 s, Bruker, Billerica, MA, USA) was used to identify and quantify the phases formed. The compacts density (He Pycnometer, Accupyc II 1340, Micromeritics, Norcross, GA, USA), Brinell hardness (Emco M4U-025, Emco, Kellau, Kuchl, Austria), and tensile properties (Instron 5505) were also measured. Fractographic studies were performed using field emission scanning electron microscopy (FEGSEM, Fei Teneo, Lausanne, Switzerland) Because of the different densities of the Al matrix and the second phases appearing after a considerable amount of sintering, the absolute density of each sintered material needs to be computed. The resulting value will be considered to determine the relative density by comparing with the measured values.

## 3. Results and Discussion

### 3.1. Powder Compaction Aptitude

**Figure 1:**
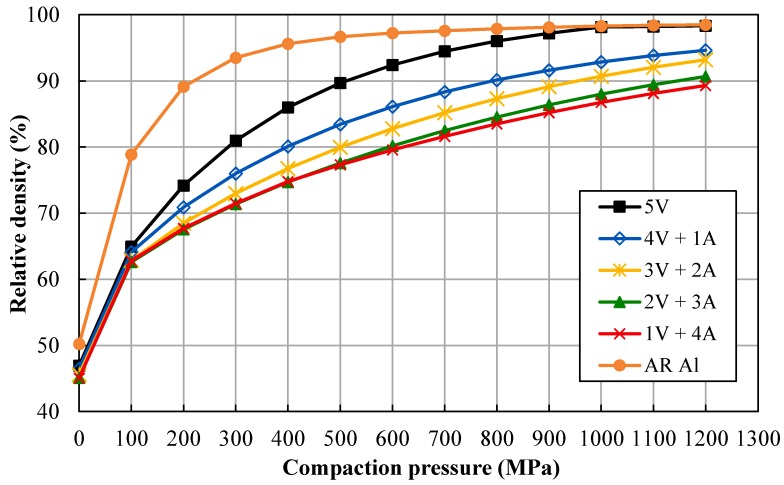
Compressibility curves of the different milled powders.

The compaction ability of the different produced powders was determined by a compressibility test, which measures the relative green density (Dg) versus the applied compaction pressure. These curves are very useful for the consolidation process, allowing determination of the adequate compaction pressure to achieve the desired green density. Compressibility curves of the different milled powders are shown in Figure 1. As can be seen, results show that the relative density of the different powders decreases for a particular applied pressure as the ammonia milling period is prolonged. Thus, Al powder milled under vacuum (V) for 5 h (5V) reaches relative densities above 90% for pressures of 500 MPa or higher. Conversely, if aluminum powder is milled for 4 h under vacuum followed by 1 h under ammonia gas flow (A; 4V + 1A), the relative density reaches values above 83% for the same pressure range. In this case, pressure must be increased up to 800 MPa in order to reach the same level of densification as that achieved for 5V powders. In the same way, for longer lasting ammonia gas flow millings (3V + 2A, 2V + 3A, and 1V + 4A), the relative density decreases, reaching values below 80% for a pressure of 500 MPa.

The compressibility test can also serve to estimate the hardness of the powder particles, showing the effect of the milling conditions. As shown in Figure 1, the relative density of the powders decreases by prolonging the ammonia gas flow, certainly because the powders become harder during the milling process. Thus, unmilled AR Al achieves relative densities higher than 90% from pressures of about 200 MPa, while vacuum-milled powder (5V) needs pressures greater than 500 MPa to achieve similar densities. The soft AR Al, therefore, hardens after milling under vacuum (5V) as a result of strain taking place during mechanical alloying. If NH_3_ is incorporated into the milling process, the achieved relative density decreases, and more so for longer intervals of NH_3_. Bearing in mind that the milling process lasted the same time for all the experiments and that the consolidation process was the same for all the powders, it is clear that the attained relative density directly depends on the milling time under ammonia gas flow.

**Figure 2:**
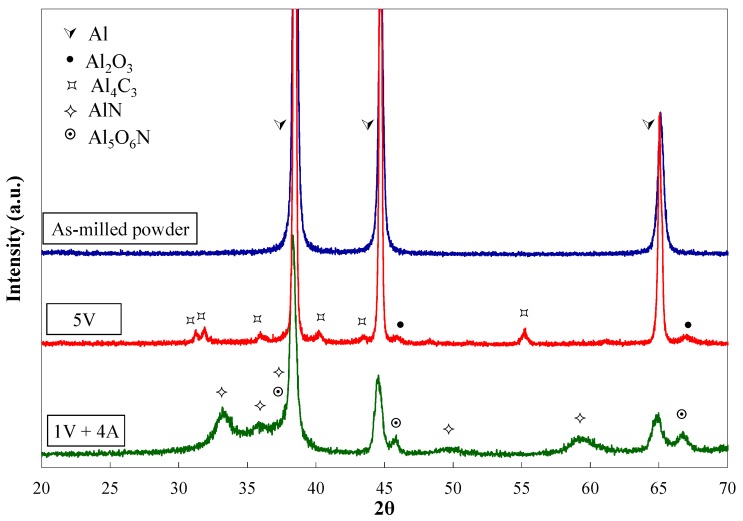
XRD patterns of as-milled powders and 5V and 1V + 4A powders after being milled and heat treated.

The XRD patterns, corresponding to all the as-milled powders, show only Al peaks (Figure 2). However, after being heated, the reflections of aluminium nitride (AlN) and alumnium oxynitride (Al_5_O_6_N) are observed besides those of the aluminium when ammonia is incorporated into the milling. This fact allows the statement that the dissociation of the ammonia gas occurs during milling, and nitrogen is kept as a solid in solution until the heat treatment activates the formation of the nitrogen-rich second phases.

### 3.2. Sintered Compact Properties

After mechanical alloying, powders were consolidated to assess the influence of the ammonia flow time on the relative density, hardness, and tensile properties.

**Figure 3:**
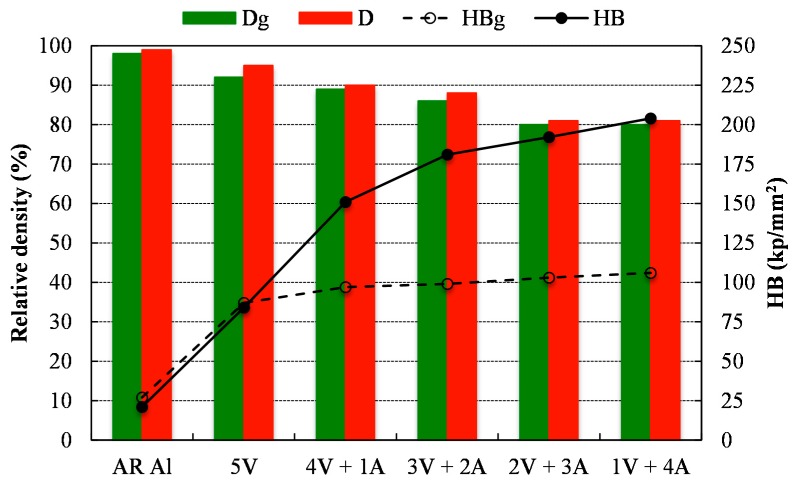
Relative density and hardness, both after pressing (D_g_ and HB_g_, respectively) and sintering (D and HB, respectively), of the different milled powders.

Figure 3 shows the relative density (D) and Brinell hardness (HB) of the different compacts after being pressed (green compacts) and sintered. It can be observed that the relative density of the 5V compacts, both before and after sintering, is slightly lower than that of the AR Al. Furthermore, relative density decreases as ammonia flow time is prolonged. The longer the ammonia flow time, the higher the hardness of the powder and as a consequence, the lower the compressibility and the achieved relative density. As expected, the relative density slightly increases for any of the studied conditions after the sintering process.

Despite the fact that the relative density follows a decreasing trend with the increase of the ammonia flow time, it should be noted that hardness follows a growing tendency. After pressing, a remarkable increase of the Brinell hardness (HB_g_) is observed for those compacts made from milled powders with respect to the AR Al compact. This increase is mainly due to strain hardening taking place during the milling process [23] as well as the resulting solid solution from the incorporation of nitrogen to the aluminum lattice [24]. After sintering, the same hardness was observed for compacts made from AR Al and 5 V powders, while compacts made from powders milled under ammonia gas show a marked increase. The reaction taking place during the sintering process between Al and N gives rise to the formation of Al_5_O_6_N and mainly, AlN [24,25]. This new phase is ceramic in nature and therefore harder than Al, therefore increasing the sintered compacts’ hardness (4V + 1A, 3V + 2A, 2V + 3A and 1V + 4A), in spite of the lower relative density achieved.

**Figure 4:**
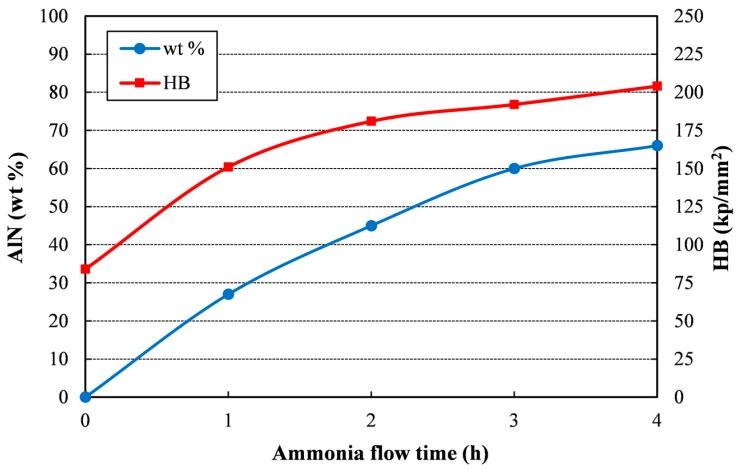
AlN wt % and hardness versus ammonia flow time of the different sintered compacts.

Figure 4 summarizes the evolution of both the weight percentage of AlN and the compact hardness. It can be observed that the amount of aluminum nitride increases with the milling time under ammonia, but the slope of the curve continuously decreases, and also does the percentage of AlN produced per hour. The direct effect on hardness of the AlN ceramic phase accounts for the similar trend observed in the hardness curve.

**Figure 5:**
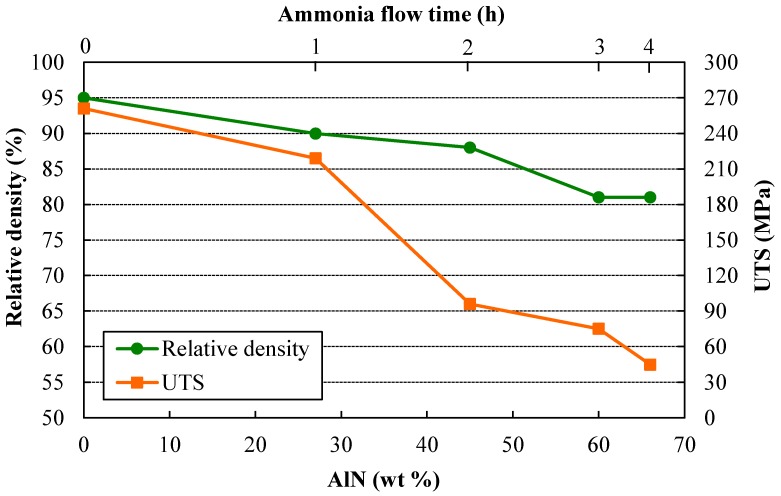
Relative density and UTS (Ultimate Tensile Strength) versus AlN wt % of the different sintered compacts.

Figure 5 shows the relative density and ultimate tensile strength (UTS) as a function of the AlN wt %. As can be observed, both properties follow the same trend, with the UTS decreasing despite the strengthening observed in hardness tests by increasing the ammonia presence (Figure 2 and Figure 3). The UTS of PM (Powder Metallurgy) parts is a function of both the relative density and the hardness of the individual powder particles, as long as good particle bonding has been achieved and the tensile test behaves in a ductile mode. On one hand, the relative density achieved after sintering decreases with the milling time under ammonia flow. Thus, the UTS tends to decrease due to the increase of porosity. Harder powders are obtained for longer millings under ammonia as a result of the solubilized N, and lower densities can be attained after pressing. On the other hand, the direct effect of the hardness of individual particles can be observed by comparing the curves in Figure 5. The UTS decreases as a consequence of the decrease in relative density, but mainly because of the increase in fragile AlN particles formed from the solubilized N. For the first hour under ammonia flow (4V + 1A), both curves are almost parallel. However, after the second hour under ammonia flow (3V + 2A), the UTS remarkably decreases due to the AlN increase from 27% to 45%. Finally, it can be observed that from the third to the fourth hour under ammonia gas (2V + 3A and 1V + 4A, respectively), the relative density no longer changes, but the UTS continues decreasing as a result of the increase in AlN from 60 to 66 wt %. Clearly, the presence of such a large amount of fragile ceramic particles controls the material’s behavior, and despite the aforementioned increase in hardness for longer millings under ammonia, the tensile response does not correspond to the expected performance in a harder ductile material.

**Figure 6:**
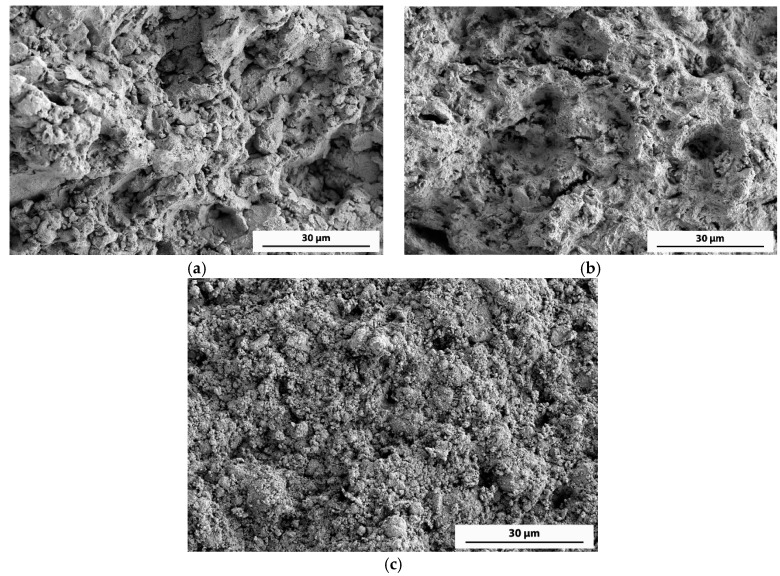
FEGSEM (field emission scanning electron microscopy) secondary electrons microfractographs of the fracture surface of (**a**) 10’A, (**b**) 4V + 1A, and (**c**) 1V + 4A compacts.

In order to reduce the high percentage of second phases, a different milling process was carried out. After two hours of milling under vacuum, a short ammonia gas flow was incorporated for ten minutes, continuing under vacuum until a total time of 5 h was reached (10’A). In this way, the presence of AlN was reduced to 19 wt %, and a relative density close to 100% was reached after the same pressing and sintering process. The resulting mechanical properties show a hardness of 167 kp/mm^2^ and a notable improvement in UTS (459 MPa). The achieved improvement is due to the high level of densification reached and the influence of an appropriate amount of ceramic particles. Figure 6 shows the fracture surface of the 10’A compact, as well as that of the 4V + 1A and 1V + 4A compacts. A low-porosity surface with rounded edges can be observed in the 10’A compact (Figure 6a), whereas the 4V + 1A compact (Figure 6b) shows a higher porosity and sharper edges. Finally, in Figure 6c is observed an even lower density surface with a plentiful presence of loose particles, although the tensile strength achieves a considerable value.

**Figure 7:**
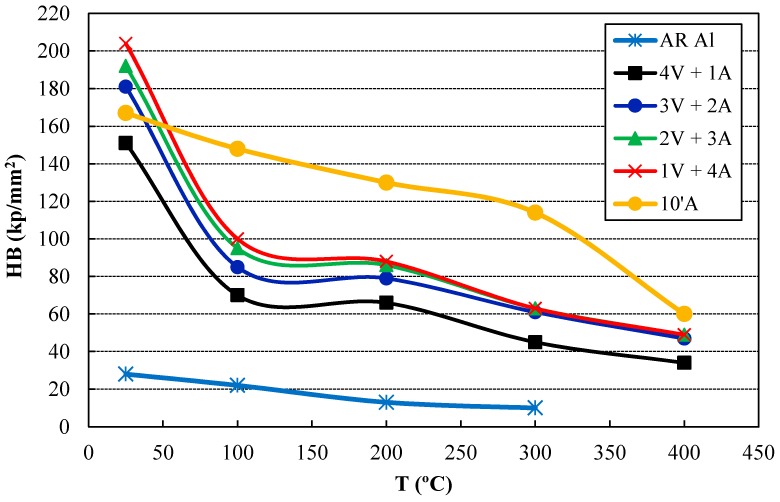
Hardness results of the sintered compacts measured at room temperature and at high temperatures after a heat treatment of 100 h at 400 °C.

Due to the high percentage of the refractory phase formed, the high-temperature behavior of the sintered compacts is expected to be noticeable. Thus, samples were heated for 100 h at 400 °C and hardness was then measured at this temperature, as well as at 300, 200, and 100 °C (Figure 7). As expected, hardness decreases as temperature increases for all samples. However, it can be observed that the hardness curves drastically decrease for long-lasting ammonia milling compacts, while it decreases more slowly for the 10’A compact as temperature rises. Furthermore, it is remarkable that the 10’A compact reaches the highest hardness values, in spite of its lower percentage of second phases. This is because the 10’A compact achieves a high-enough reinforcement of the aluminum matrix and at the same time, minimizes the porosity.

## 4. Conclusions

The as-received aluminum powder was attrition milled under different cycles of vacuum (5 Pa) and ammonia gas flow (1 cm^3^/s). Results show that in addition to strain hardening, which takes place during mechanical alloying, NH_3_ plays an additional role in powder hardening. In this way, at a consolidation pressure of 850 MPa, the relative green density is progressively reduced from about 97% to 84% (for 5V and 1V + 4A powders, respectively) when ammonia flow is prolonged, due to the increase of solubilized nitrogen. In this way, after sintering, hardness increases due to the AlN formed, while the UTS decreases as a consequence of the increase in porosity and the massive presence of fragile particles. Regarding the latter, fractographic study has revealed that a higher level of consolidation is achieved if the percentage of nitrogen-rich second phases is reduced. On the other hand, hardness tests carried out at high temperatures show that higher values are attained as the weight percentage of AlN increases. However, using an ammonia flow of 10 min, the percentage of second phases was reduced to 19% and the porosity was minimized. As a result, a value of the UTS as high as 459 MPa was reached, and the hardness at high temperatures resulted as being higher and more stable.

## Figures and Tables

**Table 1:** Milling time conditions (V = Vacuum and A = Ammonia gas flow).

Test Case	Vacuum Period (h)	NH_3_ Gas Flow Period (h)	Sample
1	5	0	5V
2	4	1	4V + 1A
3	3	2	3V + 2A
4	2	3	2V + 3A
5	1	4	1V + 4A
